# Characterizing Physical Resilience in Older Adults: Insights From Secondary Analyses in Three Clinical Groups

**DOI:** 10.1093/geroni/igaa057.2712

**Published:** 2020-12-16

**Authors:** Brian Buta, Anne Newman

## Abstract

Resilience is defined as the ability of a physiologic system to recover from a stressor that has pushed the system into a state far from its original state of equilibrium. The level of resilience can be understood by whether or not the system’s essential identity and function are retained following the stressor. The study of resilience in older adults has potential to provide clinically relevant insights into our understanding of who will or will not recover when encountering a stressful medical procedure, especially those common to older patients. The main Study of Physical Resilience and Aging (SPRING) at Johns Hopkins includes prospective data collection of determinants, phenotypes, surrogates, dynamic stimulation measures, and outcomes of resiliency among older persons undergoing knee replacement surgery, or the initiation of hemodialysis, or bone marrow transplantation. SPRING also includes analyses of existing data sources to inform these prospective studies. This symposium briefly presents the conceptual framework and design of SPRING, and focuses on the results of secondary analyses from three existing data sets that mirror the ongoing stressor studies: FORCE-TKR (knee/joint replacement, N=9006), CHOICE (dialysis initiation, N=487), and a database of patients undergoing treatment for hematologic malignancies (bone marrow transplantation, N=1011). For each clinical population, we present results on phenotypic and/or biomarker trajectories, as well as the factors associated with resilience phenotypes and how these are predictive of clinical outcomes. These analyses display the utility of resilience phenotypes for predicting risk of adverse outcomes and complement the new data being collected in our main study.

